# Functional Analysis and RNA Sequencing Indicate the Regulatory Role of *Argonaute1* in Tomato Compound Leaf Development

**DOI:** 10.1371/journal.pone.0140756

**Published:** 2015-10-19

**Authors:** Tian Wang, Rui Li, Liwei Wen, Daqi Fu, Benzhong Zhu, Yunbo Luo, Hongliang Zhu

**Affiliations:** Department of Food Biotechnology, College of Food Science and Nutritional Engineering, China Agricultural University, Beijing, China; Louisiana State University Agricultural Center, UNITED STATES

## Abstract

Regardless of whether a leaf is simple or compound, the mechanism underlying its development will give rise to a full comprehension of plant morphogenesis. The role of *Argonaute1* (*AGO1*) in the development of simple leaves has been established, but its role in the development of compound leaves remains to be characterized. In this paper, a virus-induced gene silencing (VIGS) strategy was used to dramatically down-regulate the expression of *AGO1* ortholog in tomatoes, a model plant for research into compound leaves. *AGO1*-silenced tomato compound leaves exhibited morphological defects of leaf adaxial-abaxial and trichome development. Analysis of global gene expression profiles indicated that the silencing of *AGO1* in tomato compound leaf caused significant changes in the expression of several critical genes, including *Auxin Response Factor 4* (*ARF4*) and *Non-expressor of PR5* (*NPR5*), which were involved in adaxial-abaxial formation and *IAA15* that was found to contribute to growth of trichomes as well as *Gibberellic Acid Insensitive* (*GAI*) which participated in hormone regulation. Collectively, these results shed light on the complicated mechanism by which *AGO1* regulates compound leaf development.

## Introduction

Leaf development is divided into three continuous main stages: initiation, primary morphogenesis, and secondary morphogenesis. First, the leaf arises at the flanks of the shoot apical meristem (SAM) (initiation stage). Then, the blade is initiated and forms (primary morphogenesis stage). Finally, the leaf area grows substantially, forming a leaf (secondary morphogenesis stage) [1]. Although leaves show great diversity in their shape and size, they are normally divided into two major morphogenetic groups: simple and compound. Simple leaves are each a single unit consisting of a petiole and a blade (lamina). *Arabidopsis*, tobacco, maize, and rice have simple leaves [2]. During the past decades, simple leaf development has been well characterized at the molecular level, and many genes and pathways have been found to be involved [3]. The morphogenesis stage is longer in compound leaves than in simple leaves, allowing for the initiation of leaflets [1]. The blades of compound leaves are divided into several units called leaflets, which are attached to the central leaf rachis and each resembles a simple leaf. The formation of a compound leaf requires the balance of indeterminate and determinate growth at the leaf margin to form a leaflet. In this way, compound leaf development may serve as an attractive model for the study of mechanisms underlying complex morphologies, which specified the location and formation of lateral organs. In the past, the fundamental mechanism of regulation on compound leaf development has been demonstrated at the molecular and physiological levels [1, 4, 5].

*AGO1* was first identified as a locus control of leaf development in *Arabidopsis*, and *ago1-1* null mutant exhibited severe developmental abnormalities. Because these unusual mutants look like a small squid, argonaut, they were named *argonaute* [6]. Genetic research suggested that *AGO1* may play a critical role in simple leaf adaxial-abaxial, proximal–distal, and venation patterning [7–9] and in the development of stomatal complexes [9, 10]. AGO1 is a core component of RNA-induced silencing complexes (RISCs) [11, 12]. It is responsible for the gene silencing mediated by microRNA (miRNA) and small interfering RNA (siRNA) [13]. In this way, severe simple leaf phenotype of the *ago1* mutant might be due to the disruption of miRNA or siRNA-mediated gene regulation. Mutation of *AGO1* in *Arabidopsis* has been shown to induce striking accumulation of targets of miRNA and siRNA, such as miR165/166 targets (*Homeodomain-leucine Zipper III* (*HD-ZIP III*)) [14], miR824 targets (*Agamous-like 16* (*AGL16*)) [10], and *trans*-acting siRNA (ta-siRNA) targets (*ARF3* and *ARF4*) [15]. *HD-ZIP III* genes and *ARFs* play essential roles in simple leaf development [16]. This suggests that *AGO*1 is critical to regulation of simple leaf development and that it does so mainly *via* control of levels of targets of miRNAs and siRNAs. The tomato is one of the reference systems for the study of compound leaf development [4]. There are two *AGO1* orthologs in tomatoes, *SlAGO1-1* and *SlAGO1-2* [17]. However, the lack of a tomato *ago1* mutant impedes functional understanding of *SlAGO1* in compound leaf development. Recently, the function of *SlAGO1* was indirectly investigated by *Polerovirus* silencing suppressor (P0)-mediated SlAGO1 destabilization [17]. Knockdown SlAGO1 at protein level by P0 overexpression resulted in abnormal compound leaves that had filament-like shapes [17]. However, the molecular mechanism that *SlAGO1* regulates compound leaf development remains unknown. Here, *SlAGO1* expression was directly suppressed in tomato compound leaves by VIGS, which resulted in narrow, wiry leaves. RNA sequencing (RNA-seq) profile analysis indicated that large numbers of genes were differentially expressed in *SlAGO1*-silenced leaves and some of them were involved in compound leaf morphogenesis. In addition, *SlAGO1* might play an important role in trichome formation by regulating hormone-related genes. Collectively, these results shed new light on the complicated mechanism by which *SlAGO1* regulates the development of compound leaves.

## Materials and Methods

### 2.1 Plant material and growth conditions

Tomato plants of cultivar Ailsa Craig (AC) were planted in commercial tomato-cultivated soil. All plants were grown in a greenhouse at 22°C with 75% relative humidity under a 16 h light/8 h dark cycle.

### 2.2 Cloning procedures and vector construction

For the VIGS, the tobacco rattle virus (TRV)-based vectors pTRV1 and pTRV2 were adopted. A pTRV2-*SlAGO1* recombinant plasmid was constructed by inserting a 498 bp *Eco*RI/*Eco*RI DNA fragment (*SlAGO1* fragment) into pTRV2 vector. The *SlAGO1* fragment was PCR amplified from tomato cDNA using primers *SlAGO1*-VIGS For and *SlAGO1*-VIGS Rev. Oligonucleotide primers used are listed in S3 Table.

### 2.3 VIGS treatments

*Agrobacterium tumefaciens* strain GV3101 containing pTRV1, pTRV2 or pTRV2-*SlAGO1* vectors were cultured at 28°C in LB medium (pH 5.6) containing 10 mM morpholineethanesulfonic acid and 20 μM acetosyringone with kanamycin, gentamycin, and rifampicin antibiotics. After shaking for 16 h, cultures were harvested and re-suspended in infiltration buffer (10 mM MgCl_2_, 200 μM acetosyringone) to a final optical density 600 (O.D. 600) of 1.8. Re-suspensions of pTRV1 and pTRV2 or pTRV2-*SlAGO1* were mixed at a ratio of 1:1 and allowed to sit at room temperature for 3 h. *Agrobacterium* was injected into the cotyledons of seedlings with a 1 mL syringe without a needle. Tomato seedlings injected with pTRV1 and pTRV2 served as controls. Each inoculation was carried out three times and each time six different plants were injected. When VIGS produced a visible phenotype, tomato leaf samples were collected and stored at -80°C for further analysis.

### 2.4 RNA extraction and PCR

Total RNA was isolated from TRV control and TRV-*SlAGO1* leaf samples using DeTRNa reagent (EarthOx, U.S.). The RNA concentration and purity were measured using a NAS-99 spectrophotometer (ATCGene, U.S.). The RNA integrity was checked using agarose gel electrophoresis. Genomic DNA was removed from extracted total RNA by DNase treatment. Then 2 μg total RNA was used for the first-strand cDNA synthesis using a *TransScript* One-Step gDNA Removal and cDNA Synthesis SuperMix Kit (Trans, China) with oligo(dT) primer, TRV RT primer and stem-loop RT primers. PCR was performed using EasyTaq PCR SuperMix (Trans, China) with PCR system T-100 (Bio-rad, U.S.). RT-PCR conditions were as follows: 94°C for 10 min, followed by 30 cycles of 94°C for 30 s, 55°C for 30 s and 72°C for 30 s.

Quantitative PCR (qPCR) was performed using SYBR Green PCR Master Mix with a BIO-RAD PCR System CFX96 (Bio-rad, U.S.). *SlAGO1* primers that anneal outside the region targeted for VIGS were used to ensure that only the endogenous *SlAGO1* would be detected. qPCR conditions were as follows: 95°C for 3 min, followed by 40 cycles of 95°C for 10 s and 60°C for 30 s. Changes in the fluorescence of SYBR Green were monitored automatically in each cycle, and the threshold cycle (Ct) over the background was calculated for each reaction. The relative expression levels were measured using 2^−ΔΔCt^ analysis [18]. All PCR data presented are representative of three independent experiments. *Actin* and *U6* were used as the internal controls for mRNA and miRNA, respectively. The oligonucleotide primers used in this study are listed in S3 Table.

### 2.5 RNA-seq and data processing

Total RNA was extracted from the leaves of TRV-*SlAGO1* plants and TRV control plants (two biological replicates each). The total RNA samples were treated with DNase I to degrade any possible DNA contaminants. The RNA concentration and purity were measured using a NAS-99 spectrophotometer. RNA integrity was confirmed using agarose gel electrophoresis. Then the mRNA was enriched by using oligo (dT) magnetic beads. RNA libraries with inserts approximately 250–500 bp in size were prepared (BGI, China) for 50 bp single-end sequencing on the Illumina HiSeq 2000, at a depth of ≈50 million reads per library (for statistics on read counts and percentage of mapped reads, see Table 1). All RNA-seq data for this article have been deposited at the National Center for Biotechnology Information Sequence Read Archive (http://www.ncbi.nlm.nih.gov/sra/) under accession number SRP043486.

**Table 1:** Summary of read counts and percentage of mapped reads.

Library	Raw reads	Total Mapped Reads	Perfect Mapped Reads	Unique Mapped Reads
TRV-*SlAGO1*-1	58,577,976(100%)	43,421,744(74.13%)	33,974,546(58.00%)	41,463,159(70.78%)
TRV-*SlAGO1*-2	58,409,202(100%)	42,939,331(73.51%)	36,609,869(62.68%)	41,017,409(70.22%)
TRV-1	50,565,659(100%)	37,656,833(74.47%)	29,560,551(58.46%)	36,080,591(71.35%)
TRV-2	61,363,163(100%)	45,747,071(74.55%)	39,167,949(63.83%)	43,850,729(71.46%)

1, 2, biological replicates.

RNA-seq reads were checked for quality and trimmed to removing barcode and adaptor sequences. To rule out rRNAs and tRNAs, all reads were aligned to plant rRNA and tRNA sequences using the Short Oligonucleotide Analysis Package (SOAP2, http://soap.genomics.org.cn/soapaligner.html). Plant rRNAs and tRNAs information were extracted from the NCBI Non-Redundant (NR) Dataset (http://www.ncbi.nlm.nih.gov/). The cleaned reads were then aligned to the tomato reference genome (SGN release version SL2.50, ftp://ftp.sgn.cornell.edu/tomato_genome) using TopHat [19]. The raw counts of aligned reads for each tomato gene model and from each library were derived using an in-house Perl script. Differentially expressed genes (DEGs) between TRV-*SlAGO1* and TRV leaf samples were identified using the DESeq package [20]. Genes exhibiting a |fold-change| ≥2 and adjusted *P*-value <0.05 were selected for DEGs.

### 2.6 Gene Ontology (GO) enrichment analysis

Because GO terms were not well annotated for tomato genes, the corresponding *Arabidopsis* best homologues were collected and the WEGO (http://wego.genomics.org.cn/) was used for GO enrichment. GO enrichment analysis provided all GO terms that significantly enriched in DEGs relative to the genome background and filtered the DEGs corresponding to biological processes, cellular components, and molecular functions.

### 2.7 Kyoto Encyclopedia of Genes and Genomes (KEGG) pathway enrichment analysis

Pathway enrichment analysis identifies enriched metabolic pathways and signal transduction pathways based on comparison of DEGs to the whole genome background. KEGG is the major public pathway-related database. DEGs were entered into KEGG for analysis (http://www.genome.jp/kegg/).

## Results

### 3.1 Repression of *SlAGO1* led to the abnormality of compound leaves

The severe growth arrest in *ago1* null mutants prevents functional characterization of *AGO1* at later stages of leaf development. VIGS is one of the most powerful tools for the analysis of key genes whose mutations cause embryonic and seedling-lethality [21]. The TRV-based VIGS vector is widely used for efficient silencing of genes in tomato plants [22–24]. Considering that there are two *AGO1* homologues in tomatoes, a DNA fragment of almost 500 bp that was found to be common to both *SlAGO1-1* and *SlAGO1-2* was cloned and inserted into pTRV2 vector, named pTRV2-*SlAGO1*. In order to silence *SlAGO1*, *Agrobacterium* cultures containing pTRV2-*SlAGO1* and pTRV1 were injected into the cotyledons of one-week-old tomato seedlings (S1 Fig). Cultures of pTRV2 and pTRV1 served as controls.

**Fig 1:**
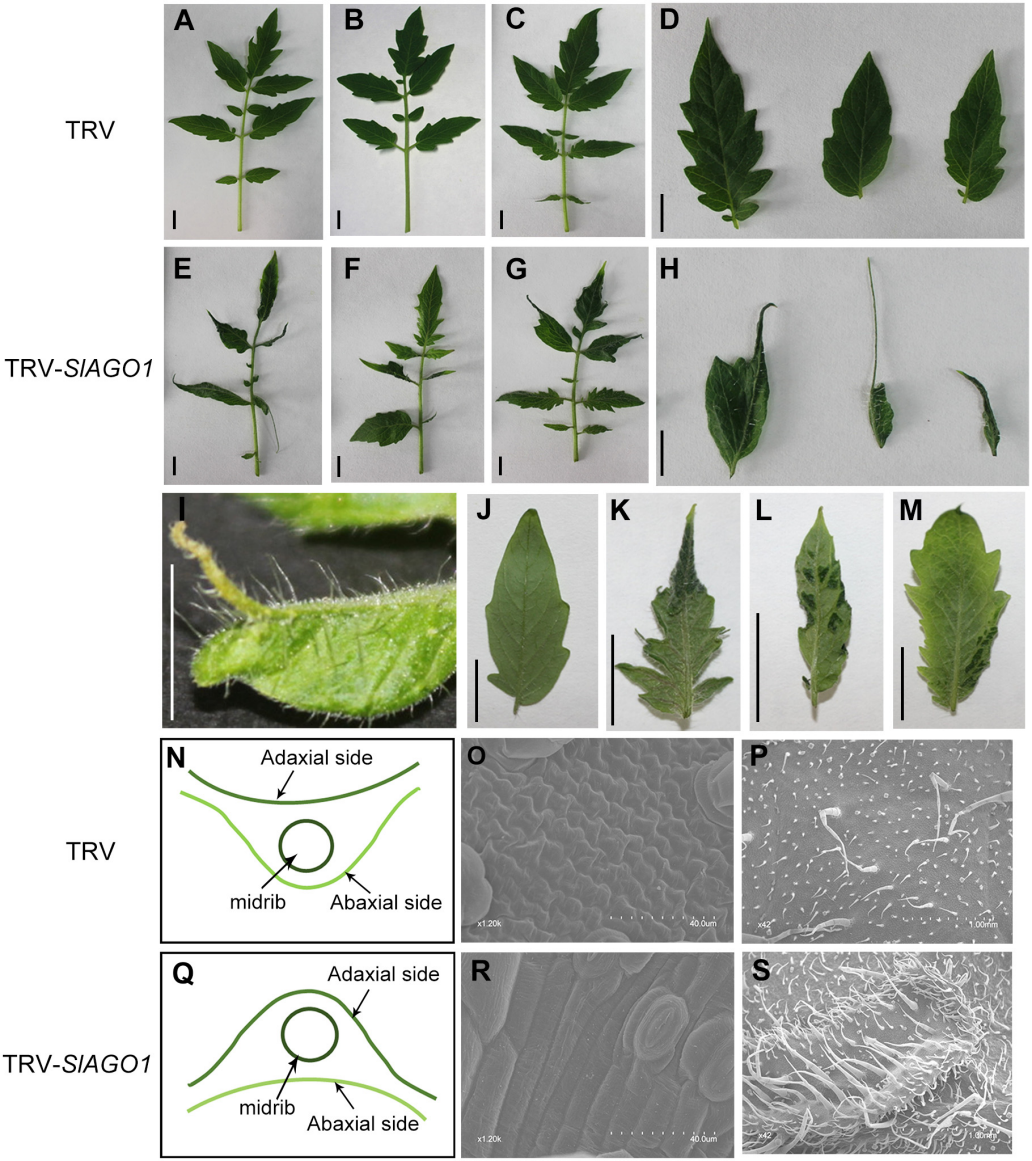
Severe phenotypes of TRV-*SlAGO1* plants. (A–C) TRV control leaf. (D) Adaxial side of TRV control leaflets. (E–G) TRV-*SlAGO1* leaf. (H, I) Adaxial side of TRV-*SlAGO1* leaflets. (J) Abaxial side of TRV control leaflets. (K-M) Abaxial side of TRV-*SlAGO1* leaflets. (N) Schematic diagram of TRV control leaflet transverse section. (O, P, R, S) Scanning electron microscopy (SEM) images of TRV control and TRV-*SlAGO1* leaflets. (O) Irregular polygon of common tomato leaf cells. (P) Glossy surface of TRV control leaflet. (Q) Schematic diagram of TRV-*SlAGO1* leaflet transverse section. (R) Columnar cells of radial leaflet. (S) Dark green patch on the abaxial side of TRV-*SlAGO1* leaflet. Scale bars = 1 cm.

**Fig 2:**
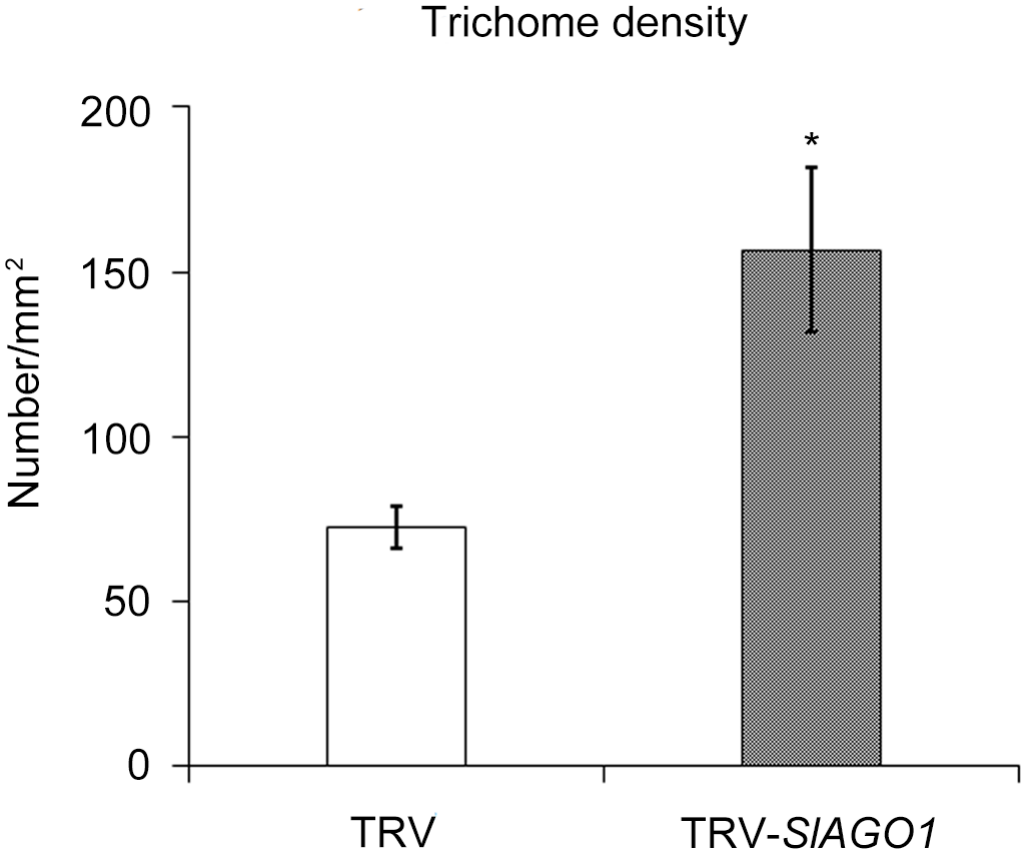
Trichome density in TRV control and TRV-*SlAGO1* plants. The trichomes in five replications of an area of 2.5 mm^2^ were counted. The error bar indicates the standard deviation of five biological replicates. Asterisks indicate significant difference as determined by student’s *t*-test (*, *P* < 0.05).

After four weeks, all the seedlings injected with TRV-*SlAGO1* exhibited a range of phenotypic abnormalities that did not shown in TRV-injected control plants. Typically, most leaflets of upper un-injected compound leaves were small, narrow, asymmetrical, and curled downward. Some of them lacked petiolules (Fig 1A–1H). Most of them lost their adaxial–abaxial polarity and had almost smooth margins (Fig 1H). Some TRV-*SlAGO1* leaflets were nearly radial and resembled shoestrings, and their midribs were outgrown or detached (Fig 1H and 1I). Moreover, dark green flecks appeared on the abaxial side of some tomato leaflets which were injected with TRV-*SlAGO1* (Fig 1K–1M). In addition, midribs of TRV control leaflets were concave on the adaxial surface whereas those of TRV-*SlAGO1* were concave on the abaxial surface (Fig 1N and 1Q). Observed under a scanning electron microscope (SEM), cells of the “shoestring” leaf were columnar in shape, resembling those of a midrib and were distinct from the irregular polygon of TRV control leaf cells (Fig 1O and 1R). SEM analysis also showed that dark green fleck had denser trichomes and glossier surface, which more closely resembled the adaxial side (Fig 1P and 1S, Fig 2).

**Fig 3:**
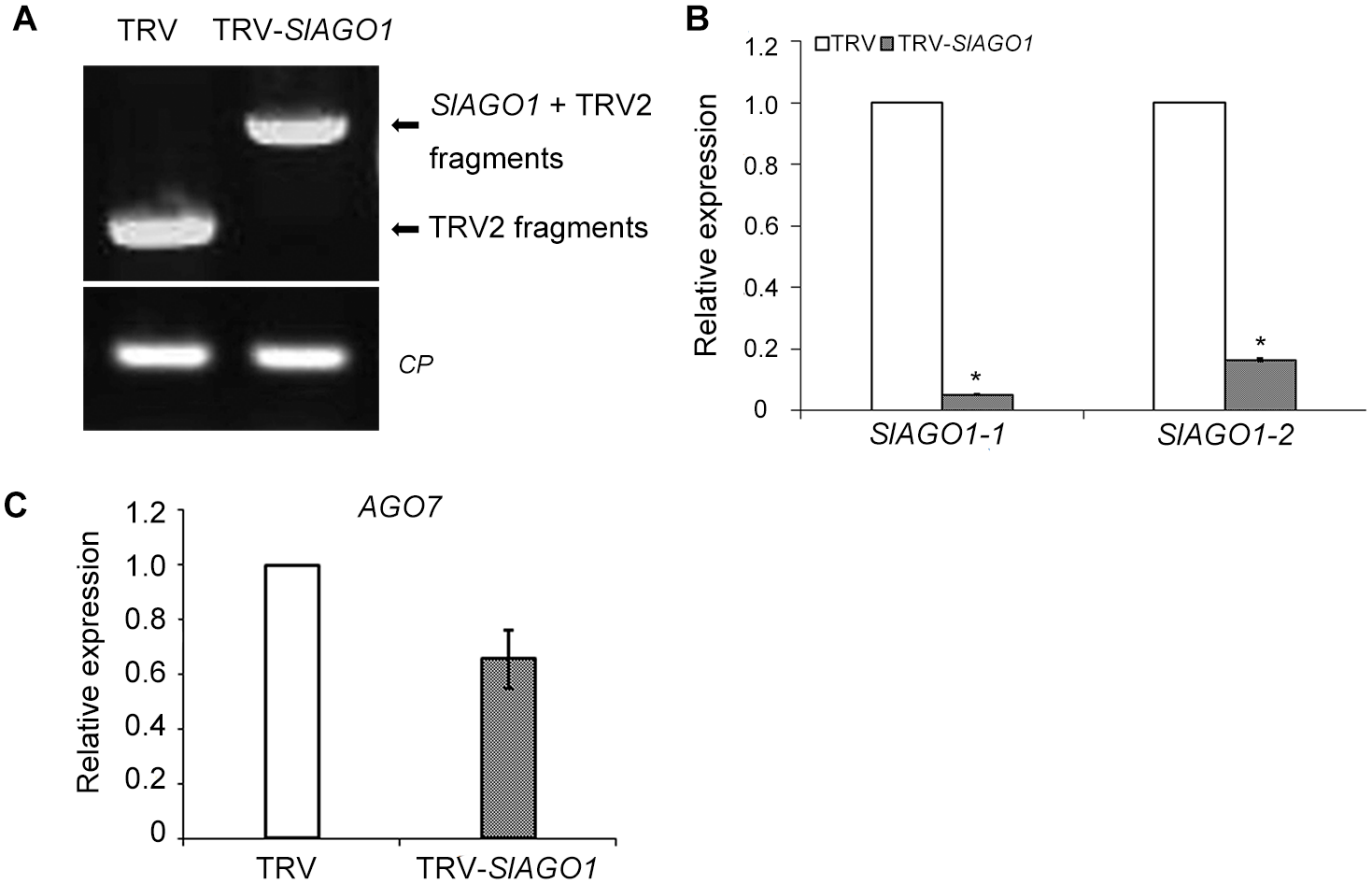
RT-PCR and qPCR analysis of TRV control and TRV-*SlAGO1* plants. (A) RT-PCR detection of TRV RNA in upper un-injected leaves. (B) qPCR analysis of *SlAGO1-1* and *SlAGO1-2* levels in plants infected with TRV control and with TRV-*SlAGO1*. (C) qPCR analysis of *AGO7* levels in TRV control and TRV- *SlAGO1* plants. The error bar indicates the standard deviation of three biological replicates. Asterisks indicate significant differences as determined by the student’s *t*-test (*, *P* < 0.01).

Since most upper un-injected leaves showed severe phenotype, suggesting that the recombinant TRV-*SlAGO1* virus moved from cotyledons to upper leaves and induced gene silencing. TRV2 specific primers were used for both TRV control and TRV-*SlAGO1* plants. The different sizes of amplified fragments clearly indicated that both TRV and recombinant TRV-*SlAGO1* can efficiently replicate and spread systemically in tomato plants and that the abnormal leaves were caused by TRV-*SlAGO1* and not TRV itself (Fig 3A). qPCR analysis indicated that levels of *SlAGO1-1* and *SlAGO1-2* were suppressed by 95% and 84%, lower in TRV-*SlAGO1* than in the TRV control, respectively (Fig 3B). Recently, impairing the biogenesis of ta-siRNAs and misregulating *ARF3* and *ARF4* in tomato *ago7* mutant was found to cause a gradient of leaf-shape aberrations, such as shoestring leaves lacking leaf blade expansion [25], resembling the TRV-*SlAGO1* phenotypes. However, the *SlAGO7* transcript levels in TRV control and TRV-*SlAGO1* injected plants were comparable (Fig 3C). Collectively, these results indicate that the specific silencing of *SlAGO1* by VIGS was responsible for the abnormal phenotype of tomato compound leaves.

### 3.2 Global overview of RNA-seq profiles of *SlAGO1*-silenced compound leaf

**Fig 4:**
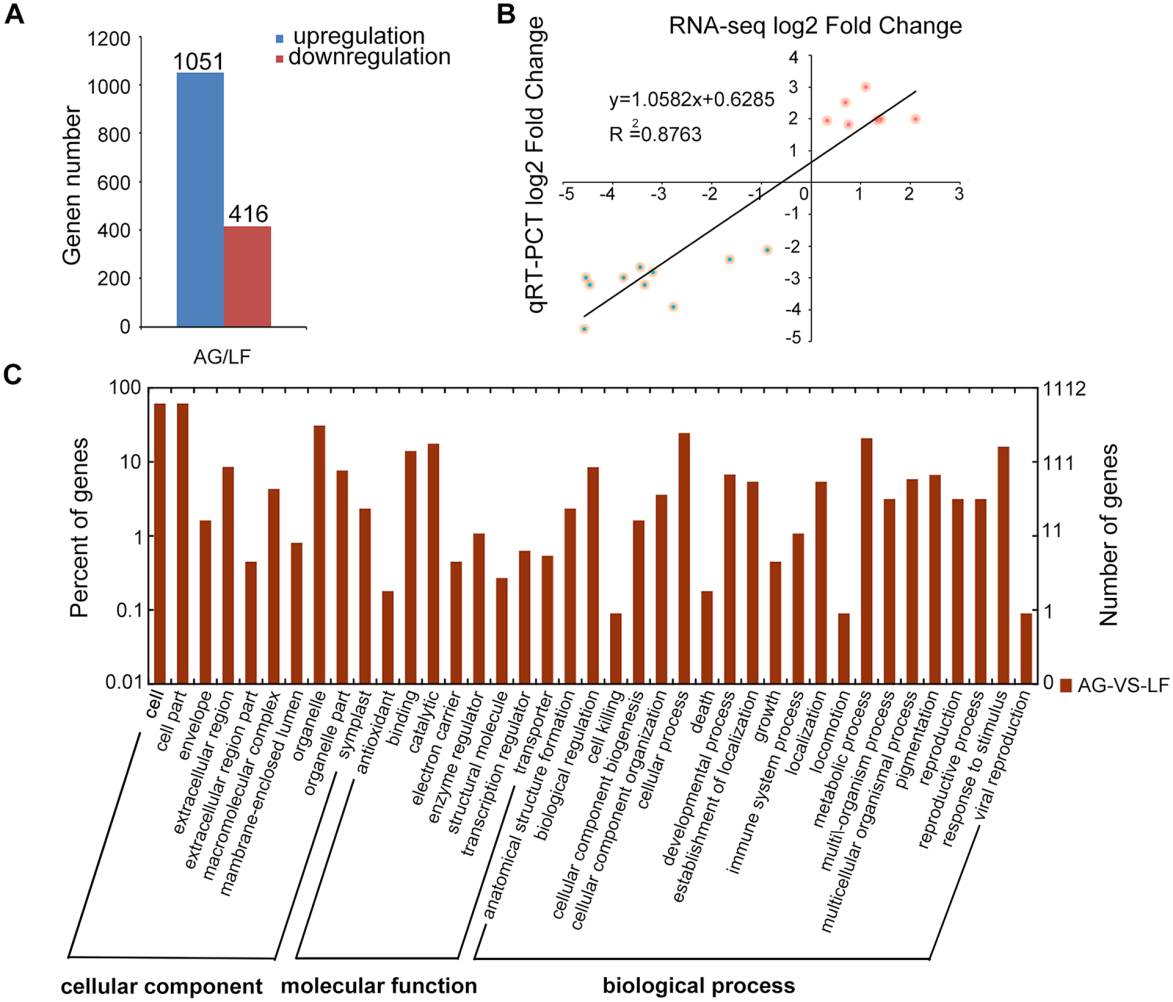
Global view of DEGs in *SlAGO1* silenced leaves. (A) The number of up-regulated and down-regulated genes in TRV- *SlAGO1* plants compared with TRV control plants. (B) Expression levels of 17 genes as determined by RNA-seq and qPCR are closely correlated. The logarithm of fold change values in the RNA-seq and the qPCR data were plotted along with the linear fit line to examine the correlation relationship between the two methods (R^2^ = 0.8763, *P* < 0.001). Red dots, up-regulated genes (Solyc02g070430, Solyc12g042890, Solyc09g072700, Solyc08g062210, Solyc05g050130, Solyc06g075650, Solyc11g040340). Blue dots indicate down-regulated genes (Solyc02g067730, Solyc02g086850, Solyc04g071400, Solyc05g015800, Solyc05g047680, Solyc07g066330, Solyc08g083500, Solyc11g008640, Solyc04g058100, Solyc11g070150). (C) GO analysis classified DEGs in TRV and TRV- *SlAGO1* tomato leaves. The functional assignments in the cellular component, molecular function, and biological process categories are shown with respect to number of genes.

To gain insights into the molecular impact of *SlAGO1* silencing on compound leaf development, gene expression patterns of *SlAGO1*-silenced leaves and TRV control leaves were compared using RNA-seq. Two RNA libraries, representing two biological replicates, were prepared and sequenced, generating more than 50 million raw reads, respectively (Table 1). In TRV control and TRV-*SlAGO1* plants, a total of 22,773 and 23,116 tomato genes, respectively, were found to be expressed. DESeq analysis showed 1,467 genes to be significantly differentially expressed between TRV-*SlAGO1* and the TRV control. Compared with TRV control, 1,051 genes were up-regulated in TRV-*SlAGO1*, and 416 genes showed dramatic down-regulation (Fig 4A, S1 Table). To determine whether RNA-seq data were reliable, 17 of the DEGs were randomly selected and their expression levels were checked using qPCR. The fold change in the DEGs expression levels of qPCR and RNA-seq was very similar (R^2^ = 0.88, *P* < 0.001) (Fig 4B).

**Fig 5:**
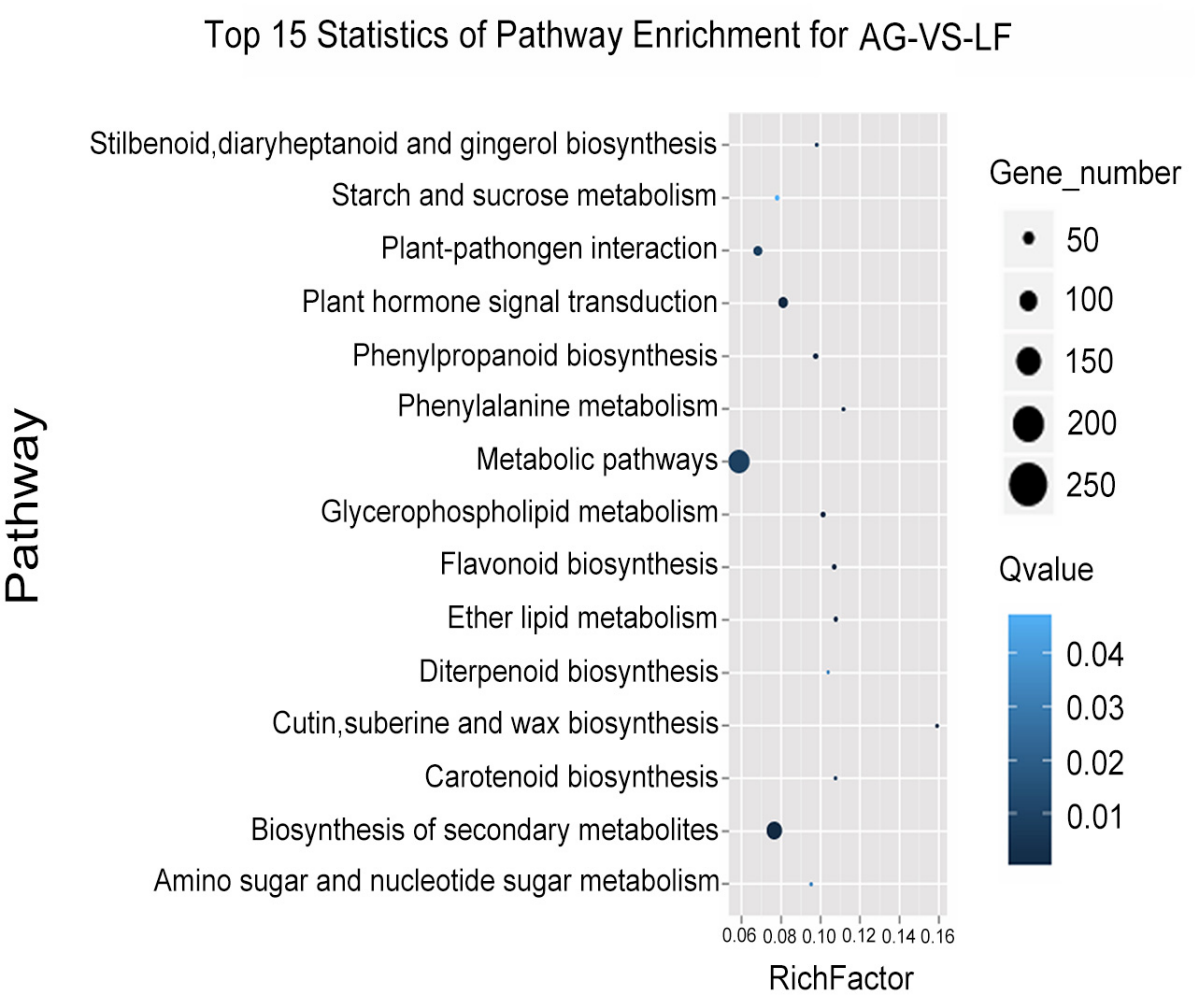
Pathway enrichment analysis of differentially displayed genes from TRV- *SlAGO1* plants.

GO analysis revealed that DEGs covered a wide range of biological processes, cellular components, and molecular function, which could be classified into 21, 10, and 8 categories. Among biological processes, the largest group was metabolic process (19.1%), followed by cellular processes (19%). The cellular component genes were placed mainly in the cell category (29%) and cell part category (29%). In terms of the number of genes, the two largest groups within the molecular function category were catalytic (45%) and binding (36%), making up four-fifths of all the DEGs (Fig 4C). Pathway enrichment analysis was accomplished using the Kyoto Encyclopedia of Genes and Genomes (KEGG), aiming at collecting genes involved in the same biological pathway. These DEGs were involved in as many as 109 pathways, of which the top 15 were shown in Fig 5, including metabolic pathways, biosynthesis of secondary metabolites, and plant hormone signal transduction.

### 3.3 *SlAGO1* influenced expression pattern of tomato genes involved in polarity development

**Fig 6:**
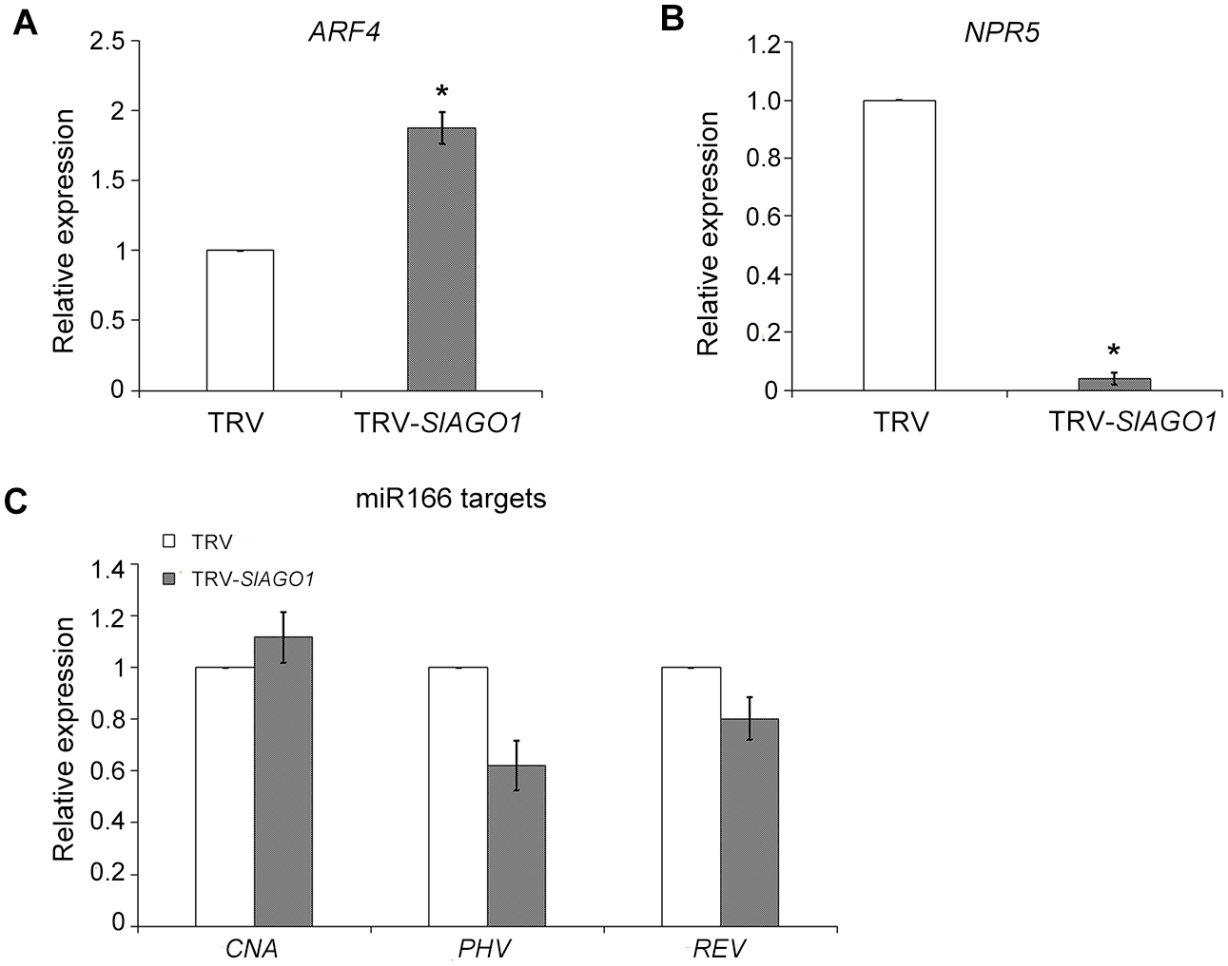
qPCR analysis of genes related to leaf development. (A) *ARF4* showed higher expression levels in *SlAGO1*-silenced leaves than in controls. (B) *NPR5* were down-regulated in TRV-*SlAGO1* control leaves. (C) target genes of miR166 displayed no significant changes when injected with TRV-*SlAGO1*. The error bar indicates the standard deviation of three biological replicates. Asterisks indicate significant difference as determined using the student’s *t*-test (*, *P* < 0.01).

Because TRV-*SlAGO1* leaves showed severe adaxial-abaxial abnormalities (Fig 1) and *AGO1* was responsible for small RNA-induced gene silencing, it is here hypothesized that the expression levels of a series of miRNA and siRNA target genes, which were involved in the determination of leaf polarity, might be affected by *SlAGO1* knockdown. *ARF4* is the target of ta-siRNA3, which participates in construction of the abaxial sides of leaves [26]. *ARF4* expression was higher in *SlAGO1*-silenced plants than in TRV control plants (Fig 6A).

Because the polarity disorder phenotype of our TRV-*SlAGO1* plants was reminiscent of rachis-like structure of *Arabidopsis BLADE-ON-PETIOLE (bop)* mutant in which *BOP* was found to participate in polarity development [27], the transcription level of *NPR5*, which is the ortholog of *BOP* in tomatoes, was checked. As expected, *NPR5* level decreased by 90% relative to the TRV control (Fig 6B).

The genes in the *HD-ZIP III* family are targets of miR166/165, including *REVOLUTA* (*REV*), *CORONA* (*CNA*), *PHAVOLUTA* (*PHV*), *PHABULOSA* (*PHB*) and *HOMEOBOX GENE 8* (*HB8*), which govern the formation of the adaxial side of simple leaves. In *Arabidopsis ago1* mutants, *PHB*, *PHV*, and *REV* expression levels are increased throughout the leaf [8, 28]. Surprisingly, no significant differences in the accumulation of *PHV* and *REV* were observed between *SlAGO1*-silenced tomato plants or in controls plants (Fig 6C), suggesting that the regulation mechanism of *HD-ZIP III* family in compound leaf development remained to be characterized.

**Fig 7:**
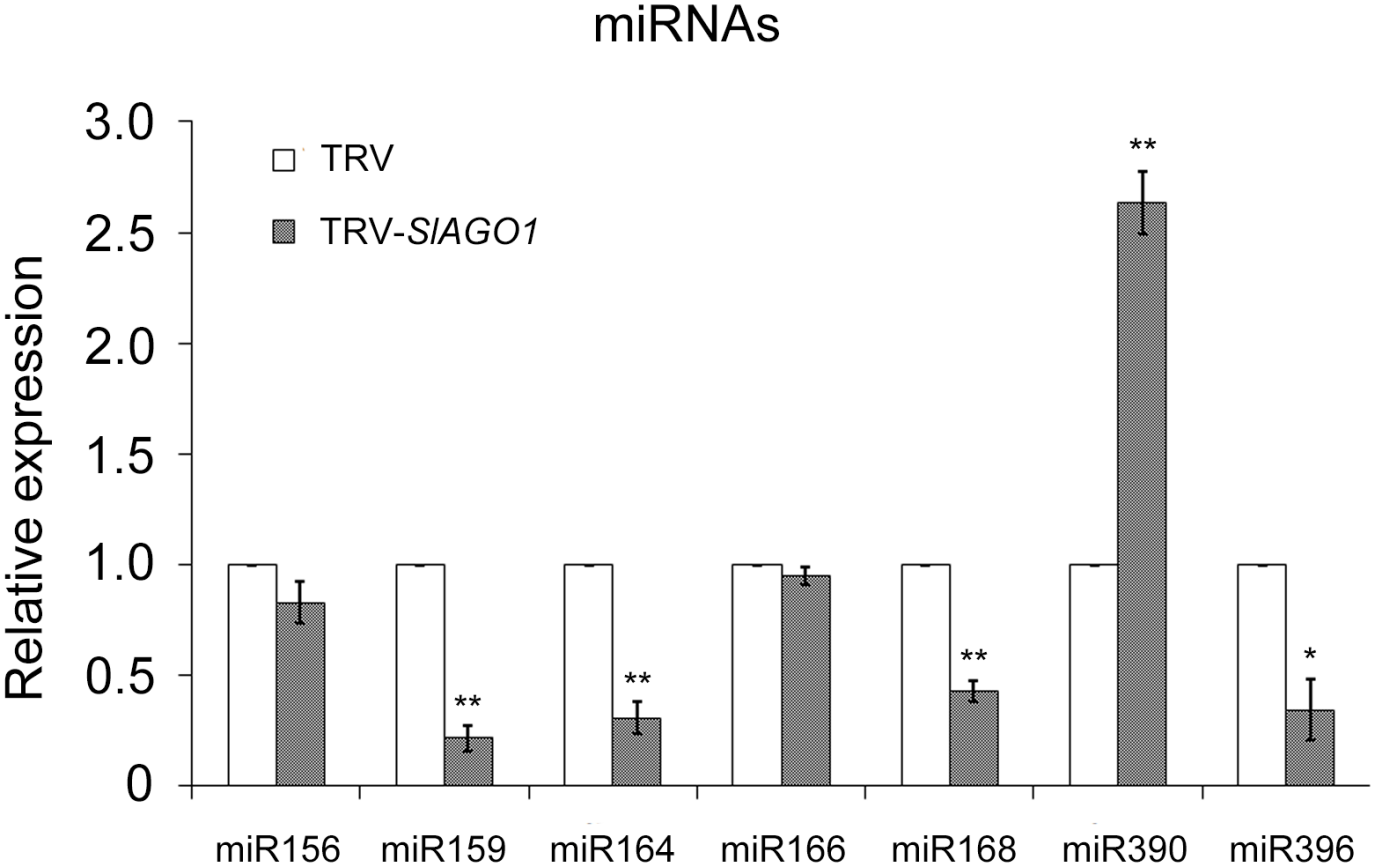
qPCR analysis of miRNA accumulation. The relative expression of miRNAs showed various changes. The error bar indicates the standard deviation of three biological replicates. Asterisks indicate significant difference as determined using the student’s *t*-test (*, *P* < 0.05, **, *P* < 0.01).

The accumulation of mature miRNA was also analyzed (Fig 7). Their relative expressions showed various changes. miR159, miR164, miR168, and miR396 declined significantly and miR390 rose visibly. miR156 and the abovementioned miR166 exhibited no visible changes. One possible reason for this might be that AGO1 was involved in miRNA-mediated RNA degradation and did not participate in miRNA biogenesis directly. In this way, miRNAs displayed diverse patterns of expression in *SlAGO1*-silenced leaves.

### 3.4 Genes involved in trichome development are affected in *SlAGO1*-silenced lines

**Fig 8:**
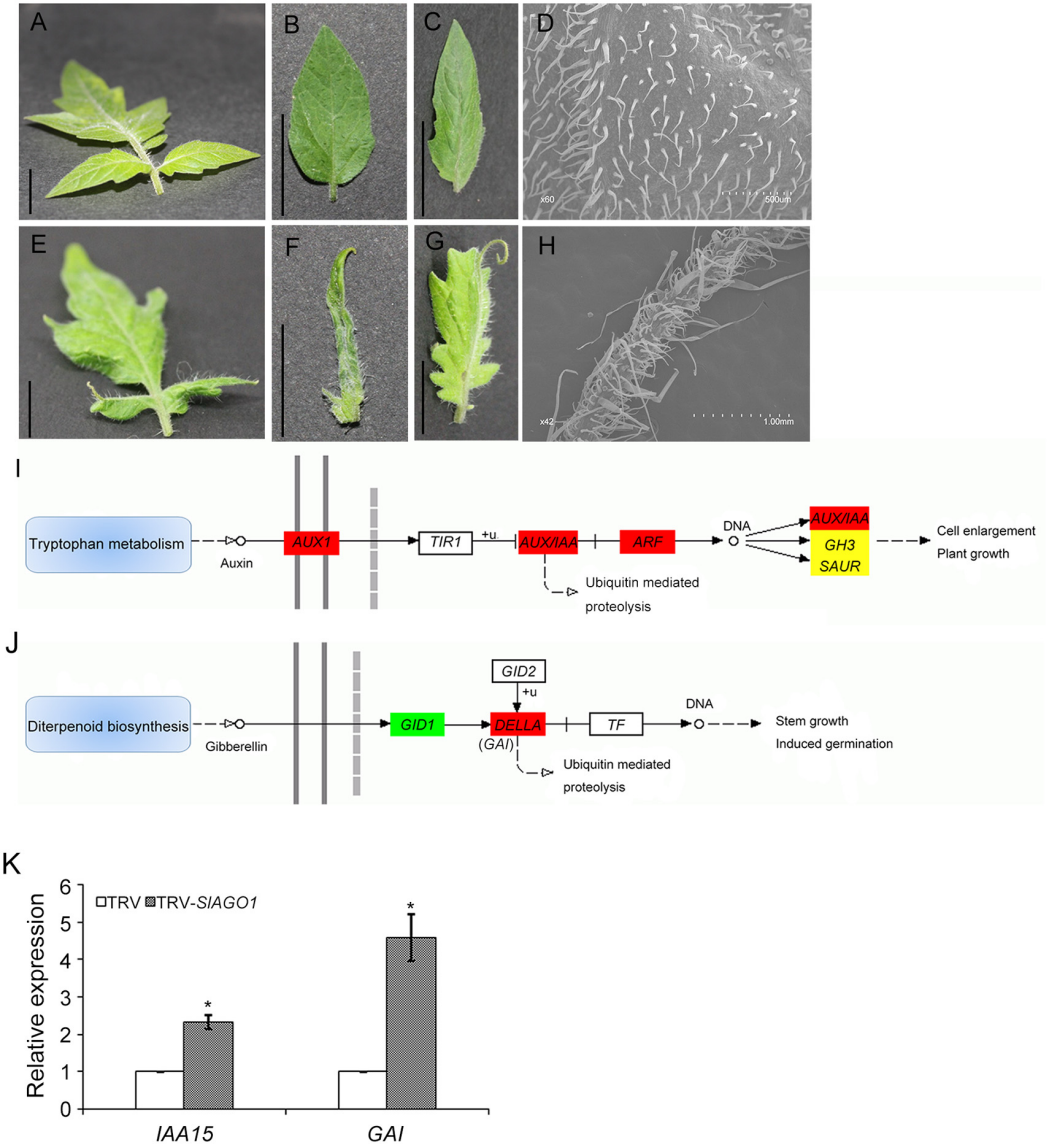
Effects of trichome development in *SlAGO1*-silenced tomato plants. (A–C) Leaflets from TRV plants. (D) SEM images of leaflets surface from TRV plants. (E–G) Leaflets from TRV-*SlAGO1* plants. (H) SEM images of leaflets surface from TRV-*SlAGO1* plants. (I, J) Schematic diagram of auxin and GA signal transduction pathways. It was constructed based on the RNA-seq data using KEGG pathway mapping. The red, yellow, and green squares indicate up-regulation, absence of significant differences, and down-regulation, respectively. +u, ubiquitination. (K) qPCR analysis of *IAA15* and *GAI* expression patterns in TRV and TRV-*SlAGO1* plants. The error bar indicates the standard deviation of three biological replicates. Asterisks indicate significant difference as determined by student’s *t*-test (*, *P* < 0.01).

There were denser and longer trichomes on the surfaces of in *SlAGO1*-silenced leaves than on those of TRV control leaves (Fig 8A–8H). Evidence has suggested that auxin and gibberellin (GA) are involved in trichome development [29, 30]. RNA-seq and KEGG pathway analysis have suggested that many DEGs are clustered in auxin and GA metabolism when *SlAGO1* is silenced (Fig 8I and 8J). For these reasons, it is here hypothesized that the anomalous trichomes on *SlAGO1*-silenced leaves are the result of abnormal hormone response. The expression level of *IAA15*, whose down-regulation would cause the glabrous phenotype, was assessed [30]. It reached 2.3 times control levels in *SlAGO1*-silenced plants (Fig 8K). This result indicated that *SlAGO1* might affect its expression level, which was involved in trichome development. We also analyzed the expression level of *GAI*, which was involved in GA pathway [29]. The relative expression level of *GAI* was 4.6 times higher in TRV-*SlAGO1* plants (Fig 8K), suggesting that silencing of *SlAGO1* affected hormone regulation.

We made a table to compare the orthologous genes from *Arabidopsis*, rice and maize with those of tomato which were identified in this paper (S2 Table). Although, roles of all these orthologous genes from other species have not been clearly established yet, the comparison will help to identify orthologous genes of other species for controlling the processes of leaf development and hormone regulation.

## Discussion

### 4.1 *SlAGO1* can be silenced efficiently by VIGS

VIGS is widely used to identify gene function because it is rapid, reliable, transformation-free, and easy to perform [31, 32]. It can also repress endogenous genes through inoculation of a recombinant virus carrying a specific fragment of the host gene [33]. When the virus infects plants and spreads systemically throughout them, the endogenous gene transcripts, which are homologous to the insert fragment in the virus vector, are degraded by post-transcriptional gene silencing (PTGS) [34]. Mi *et al*. supposed that VIGS was compromised in the *ago1* mutant, with greatly reduced photo bleaching when injected with TRV-*PDS* [33]. However, the level of endogenous *SlAGO1* gene decreased strikingly in response to VIGS, resulting in unusually shaped leaves (Figs 1 and 2). It is here reasoned that, although *AGO1* plays pivotal roles in VIGS, other AGOs might also supplement it. For instance, AGO1 and AGO2 have redundant roles in miR408-mediated plantacyanin regulation [35]. In a recent study, Shao *et al*. used high-throughput sequencing to search small RNAs that enriched in the four AGO proteins, AGO2, AGO5, AGO7, and AGO10. They discovered that AGO2 and AGO5 might be implicated in the miRNA mediated pathway [36]. In tobacco, AGO1 and AGO4 were found to cooperate with VIGS to achieve extensive systemic silencing [37]. All of the evidence given above shows that other AGO proteins might act as substitutions of AGO1 in VIGS. The exact roles of other AGOs in VIGS remain to be determined and will require intensive study. However, it is clear that *AGO1* can be silenced by VIGS, at least in tomato plants. This sheds new light on the rapid and functional analysis of AGO family by VIGS.

### 4.2 Common factors played distinguish roles between simple and compound leaf polarity

Morphogenesis of simple and compound leaves is regulated by distinct genetic mechanism with shared common key factors [4, 38]. *Class1 KNOTTED1-LIKE HOMEBOX* (*KNOX1*) transcription factor is one of most important factors for SAM development in *Arabidopsis* and maize, whose expression is restricted to the SAM [39]. However, in tomato plants, the *KNOXI* genes *Tomato KNOTTED1* (*TKN1*) and *TKN2* expression is restored in developing primordial leaves [40]. This mainly affects tomato leaf maturation [41].

In *Arabidopsis*, genes of the *HD-ZIP III* family are critical to adaxial-abaxial formation, which are targets of miR166/165 [14]. Overexpression of *CNA* resulted in radialized and trumpet-shaped leaves in *Arabidopsis* [42]. Both gain-of-function mutants *phb* and *phv* led to leaf polarity defects and the formation of meristems in ectopic positions [43], and similar mutations in the *REV* gene caused polarity disorders in leaves [44]. In this study, *SlAGO1*-silenced leaves showed disorganized adaxial-abaxial sides, or even loss of polarity, failing to form a blade and developing with radial symmetry (Figs 1H and 8F). However, surprisingly, the transcription expression of *HD-ZIP III* family genes were not increased in the case of *SlAGO1* knockdown but rather unaffected (Fig 6C). There have been few reports that focused on the functions of *HD-ZIP III* family in tomato leaf construction so far. Hu *et al*. overexpressed *REV* in tomato and the plants produced leaves whose edges curled upward [45], but there was no effect on polarity development. P0-mediated SlAGO1 degradation did not result in any change in the level of *REV* expression [17]. This was consistent with current results showing that REV had no visible differences in TRV-SlAGO1 leaves relative to TRV control leaves (Fig 6C). It is possible that *SlAGO1* might regulate *HD-ZIP III* family genes by translation inhibition [46].

### 4.3 Diverse regulation of *SlAGO1* on target genes of small RNA

AGO1 is involved in the regulation of gene expression *via* RISCs [47]. In general, target genes of small RNAs, such as *ARF4*, the target of ta-siRNA, are up-regulated in *SlAGO1*-silenced plants, in which *SlAGO1* acts as a negative regulator (Fig 5). The accumulation of small RNAs may contribute to disorganized polarity in TRV-*SlAGO1* leaves. There are also many miRNA target genes that manifesting no significant change in *SlAGO1*-silenced plants (S2 Fig). These results were consistent with those reported by Martienssen et al., who found that only 1 to 6% of genes displayed significant expression changes in *Arabidopsis ago1* mutant in microarrays, in which several known miRNA targets increased remarkably, whereas others showed little or no change [48]. *Growth Regulating Factor 1* (*GRF1*), which was regulated by miR396 and may reach higher levels in *SlAGO1*-silenced plants showed a marked decrease (S1 Fig) [33]. The phenotype of TRV-*SlAGO1* leaflets was similar with those of *GRFs* loss-of-function mutants, which were smaller than control ones [49]. For this reason, it is here speculated that *GRF1* might be controlled by other factors that played more substantial roles than *SlAGO1*. In addition, AGO1 might partially mediate translational repression of some of the targets of small RNAs [50].

## Supporting Information

S1 Fig Schema of VIGS treatment in tomato cotyledon. Here, 498 bp of *SlAGO1* fragment was placed in the vector of pTRV2. The plasmids of pTRV1, pTRV2-*SlAGO1* were transferred into *Agrobacterium* strain GV3101 separately, mixed at a 1:1 ratio, and injected into the cotyledon of tomato. LB: left border of T-DNA; CP: TRV coat protein; Rz: self-cleaving ribozyme; RdRP: RNA-dependent RNA polymerase; MP: movement protein; 16K: 16 kD cysteine-rich protein; RB: right border of T-DNA. The location of TRV2 specific primers is also shown.
(TIF)

S2 Fig qPCR analysis of miRNA-target transcripts. Some miRNA target genes showed no significant differences from inTRV-*SlAGO1* plants. The error bar indicates the standard deviation of three biological replicates.
(TIF)

S3 Fig qPCR analysis of *GRF1*. The error bar indicates the standard deviation of three biological replicates. Asterisks indicate significant difference as determined by the student’s *t*-test (*, *P* < 0.01).
(TIF)

S1 Table Differentially expressed genes of RNA-seq. (XLSX)

S2 Table Orthologous genes from tomato, *Arabidopsis*, rice and maize. (XLSX)

S3 Table Oligonucleotide primers used in the study. (XLSX)
