# Supplementary figures and images for: Functional Analysis and RNA Sequencing Indicate the Regulatory Role of Argonaute1 in Tomato Compound Leaf Development
Source: PLoS One. 2015 Oct 19;10(10):e0140756. doi: 10.1371/journal.pone.0140756 (PMC4610667; doi:10.1371/journal.pone.0140756)

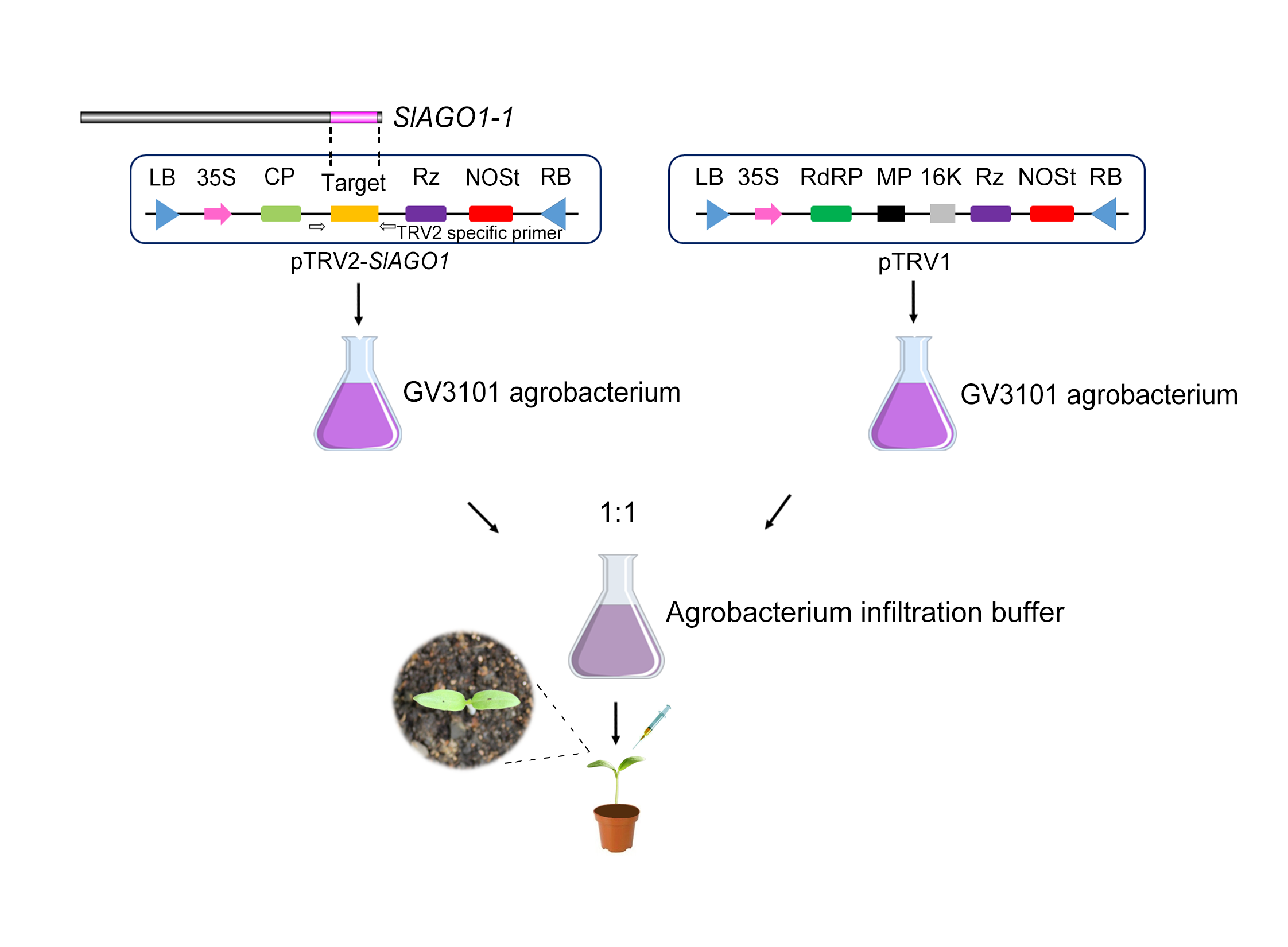

Supplement: S1 Fig — Here, 498 bp of SlAGO1 fragment was placed in the vector of pTRV2. The plasmids of pTRV1, pTRV2-SlAGO1 were transferred into Agrobacterium strain GV3101 separately, mixed at a 1:1 ratio, and injected into the cotyledon of tomato. LB: left border of T-DNA; CP: TRV coat protein; Rz: self-cleaving ribozyme; RdRP: RNA-dependent RNA polymerase; MP: movement protein; 16K: 16 kD cysteine-rich protein; RB: right border of T-DNA. The location of TRV2 specific primers is also shown. (TIF) [file pone.0140756.s001.tif]

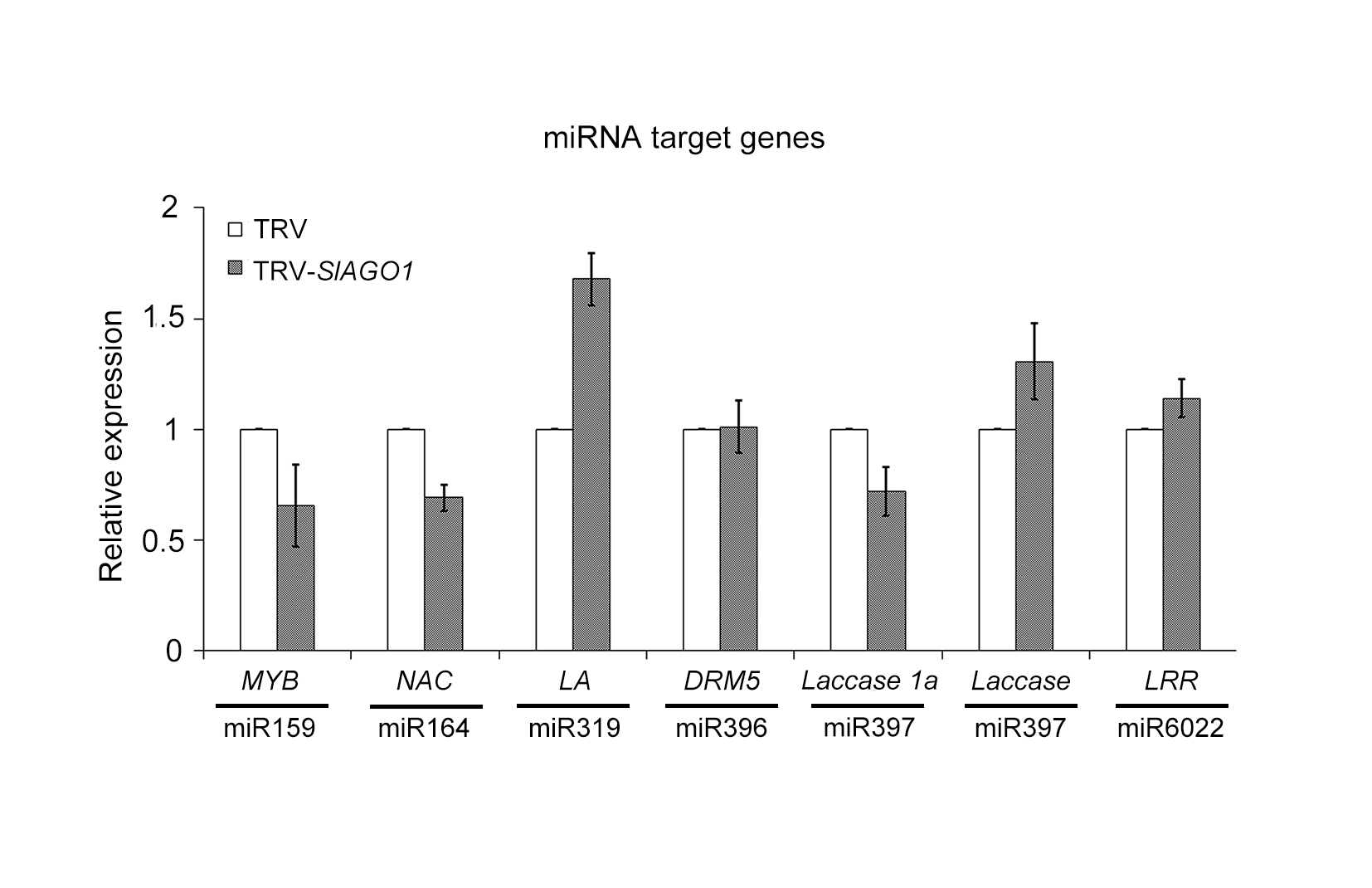

Supplement: S2 Fig — Some miRNA target genes showed no significant differences from inTRV-SlAGO1 plants. The error bar indicates the standard deviation of three biological replicates. (TIF) [file pone.0140756.s002.tif]

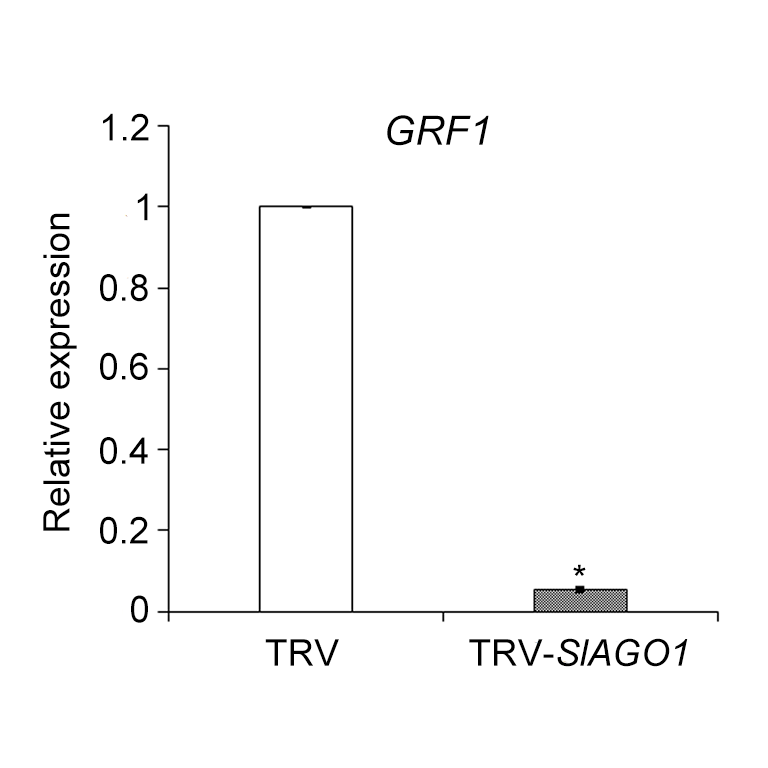

Supplement: S3 Fig — The error bar indicates the standard deviation of three biological replicates. Asterisks indicate significant difference as determined by the student’s t-test (*, P < 0.01). (TIF) [file pone.0140756.s003.tif]
